# Paradoxical Regulation of Hypoxia Inducible Factor-1α (HIF-1α) by Histone Deacetylase Inhibitor in Diffuse Large B-Cell Lymphoma 

**DOI:** 10.1371/journal.pone.0081333

**Published:** 2013-11-27

**Authors:** Savita Bhalla, Andrew M. Evens, Sheila Prachand, Paul T. Schumacker, Leo I. Gordon

**Affiliations:** 1 Department of Medicine, Division of Hematology/Oncology Northwestern University Feinberg School of Medicine and the Robert H Lurie Comprehensive Cancer Center, Chicago, Illinois, United States of America; 2 Division of Hematology/Oncology, Tufts University School of Medicine and Tufts Cancer Center, Boston, Massachusetts, United States of America; 3 Department of Pediatrics, Feinberg School of Medicine, Northwestern University, Chicago, Illinois, United States of America; University of Colorado Denver, United States of America

## Abstract

Hypoxia inducible factor (HIF) is important in cancer, as it regulates various oncogenic genes as well as genes involved in cell survival, proliferation, and migration. Elevated HIF-1 protein promotes a more aggressive tumor phenotype, and greater HIF-1 expression has been demonstrated to correlate with poorer prognosis, increased risk of metastasis and increased mortality. Recent reports suggest that HIF-1 activates autophagy, a lysosomal degradation pathway which may promote tumor cell survival. We show here that HIF-1α expression is constitutively active in multiple diffuse large B cell lymphoma (DLBCL) cell lines under normoxia and it is regulated by the PI3K/AKT pathway. PCI-24781, a pan histone deacetylase inhibitor (HDACI), enhanced accumulation of HIF-1α and induced autophagy initially, while extended incubation with the drug resulted in inhibition of HIF-1α. We tested the hypothesis that PCI-24781- induced autophagy is mediated by HIF-1α and that inhibition of HIF-1α in these cells results in attenuation of autophagy and decreased survival. We also provide evidence that autophagy serves as a survival pathway in DLBCL cells treated with PCI-24781 which suggests that the use of autophagy inhibitors such as chloroquine or 3-methyl adenine in combination with PCI-24781 may enhance apoptosis in lymphoma cells.

## Introduction

Hypoxia Inducible Factor-1 (HIF-1) is a basic helix-loop-helix transcription factor that is expressed in most cells in response to hypoxia. HIF-1 is a heterodimeric protein consisting of HIF-1α and HIF-1β subunits [1]. Under normoxic conditions, HIF-1α protein exhibits a very short half-life [2] and is rapidly degraded by the ubiquitin proteasome pathway [3,4], minimizing HIF-1 activity. In hypoxia, HIF-1α is stabilized and forms a complex with HIF-1β that allows HIF-1 to function as a transcription factor. Thus, HIF-1α is primarily activated during hypoxia under normal physiologic conditions. By contrast, HIF-1α is frequently activated in cancer cells, including under normoxic conditions by oncogene products [5] or impaired activity of tumor suppressor genes [6]. HIF-1 promotes cancer cell growth and survival and HIF gene products protect cancer cells from chemotherapeutic agents. Constitutive expression of HIF-1α has been reported in several solid tumors [7] as well as in hematologic malignancies [8,9] and elevated HIF levels have been linked to poor prognosis [7]. Gene expression profiling studies have shown that increased expression of transcription factor Hypoxia Inducible Factor-1 alpha (HIF-1α) plays an important role in the pathogenesis of Diffuse large B cell lymphoma (DLBCL) [9-11]. DLBCL is the most common aggressive form of non-Hodgkin’s lymphoma (NHL), comprising approximately 30% of all NHL [12]. Given the role of HIF in cancer, the development of agents that inhibit HIF is of great importance. A number of novel small molecule inhibitors of HIF have been identified [13-15], and various other agents have been found to exhibit HIF inhibitory activity. For example, histone deacetylase inhibitors (HDACIs) have been reported to suppress HIF-1α and the expression of HIF-regulated genes [16-18]. 

HDACIs are well-characterized anti-cancer agents with promising results in clinical trials. HDACIs mostly induce tumor cell cytostasis and apoptosis in various hematologic [19,20] and solid malignancies [21]. Different mechanisms of HDACI-induced apoptosis in cancer cells have been proposed. However, despite the promising results in clinical trials the precise mechanism of action of these inhibitors in human malignancies is still unclear. Elucidating the molecular mechanism of HIF-1α regulation by HDACI is critical in order to improve our understanding of the HIF signaling pathways and to allow the development of more specific therapies. 

HDAC inhibition has also been shown to induce autophagy [22]. Unlike apoptosis, the role of autophagy is context-dependent and it can be either cytoprotective or cytotoxic. Autophagy protects cancer cells against some anticancer treatments by blocking the apoptotic pathway (protective autophagy) while it induces cell death in others [23]. HIF-1α has been reported to play a key role in hypoxia-induced protective autophagy through BNIP3 induction [24,25]. We reasoned that if HIF-1α induces autophagy then HDACI-induced inhibition of HIF-1α should result in inhibition of autophagy. On the other hand, HDACIs have been shown to induce autophagy [26] and attenuate HIF-1α in cancer cells [26,27]. In the present study we examined these paradoxical effects of HDACI on HIF-1α and autophagy in DLBCL cells following treatment with PCI-24781, a novel pan HDACI. We sought to determine whether PCI-24781-induced autophagy is mediated by HIF-1α and whether inhibition of autophagy augments the therapeutic effect of PCI-24781 in DLBCL.

## Materials and Methods

### Ethics statement

Peripheral blood for the study was drawn from patients, after approval by the Northwestern University Institutional Review Board (IRB) and written informed consent in accordance with the declaration of Helsinki.

### Cell culture, treatment, and transfection

DLBCL (SUDHL4, SUDHL6, and OCI-LY3 and HF1) cells were grown in RPMI 1640 (Invitrogen) containing 10% or 15% (for OCI-LY3 and SUDHL6) fetal bovine serum. HDAC inhibitor PCI-24781 was provided by Pharmacyclics. Chloroquine (CQ) and 3-methyl adenine (3-MA) were purchased from Sigma. Before each assay, cells were starved overnight with 0.5% fetal bovine serum. Assays were done in 2% fetal bovine serum or as indicated otherwise.

### Primary Chronic Lymphocytic Leukemia (CLL) cells

After approval by the Northwestern University Institutional Review Board (IRB) and written informed consent in accordance with the declaration of Helsinki, peripheral blood was drawn from 3 patients with CLL. Malignant cells were purified by diluting the blood 1:1 with PBS (Ca^2+^ and Mg^2+^ free) and were layered on top of Ficoll-Paque Plus (Sigma-Aldrich). Samples were then centrifuged at 150*g* for 20 minutes at room temperature; the buffy coat layer was removed and washed with PBS twice and subsequently placed in culture with RPMI medium.

### Cell apoptosis assays

Apoptosis was measured using an apoptosis detection kit from BD Biosciences using the manufacturer’s protocol. In brief, cells were harvested washed and stained with Annexin V-FITC and propidium iodide (PI) for 15 minutes at room temperature followed by flow cytometry using a Beckman coulter XL or CyAn instruments. Data were analyzed using FCS Express software. The significance of differences between experimental conditions was determined using the Student's *t* test. 

### Western blot analysis

Cells were centrifuged, washed with cold PBS, and lysed on ice for 30 minutes in lysis buffer containing protease and phosphatase inhibitors. Protein concentrations were determined with the Bio-Rad protein assay kit (Bio-Rad, Hercules,CA). Total protein (50μg) was electrophoresed on 12% SDS polyacrylamide gels and transferred to nitrocellulose membranes, blocked for 1 hour with 50 mM Tris buffer, pH7.5 containing 0.15 M NaCl, 0.05% Tween 20 (TBST) and 5 % (wt/vol) nonfat dry milk and probed overnight at 4C with TBST containing primary antibodies. After three 10-minutes washes in TBST, the filters were incubated with horseradish peroxidase-conjugated secondary antibody in the blocking buffer for 1 hour at room temperature. After three 10 minute washes in TBST, proteins were detected by enhanced chemiluminescence detection reagents (Amersham Biosciences, Buckinghamshire, United Kingdom). HIF-1α antibody was obtained from Abcam Inc (Cambridge, MA). All other antibodies were purchased from Cell Signaling Technology, Inc. Danvers, MA. Blots were stripped and re-probed with β-actin (Santacruz Biotechnology) to use as loading control. 

### Plasmids and transfection

HIF-1α wt (plasmid no. 18949), HIF-1α mutant (P/A) (plasmid no. 18955), Myr-Akt (plasmid no. 9008) were obtained from Addgene. Cells were transfected by nucleofection using Amaxa kit V or Kit L (Lonza group). Cells were selected in neomycin for 1 week before performing any experiment.

### Lentiviral transduction

Lentivirus containing HIF-1α short hairpin RNA (shRNA) or scrambeled shRNA were obtained from Open Biosystems (Thermo Scientific). Cells (10^6^ /mL) were transduced with viral particles by spinning at 2,500 rpm in flat bottom 24 well plates for 2hr at room temperature. After 48 hr of transduction cells were selected in puromycin (6 μg/mL) for 2 weeks and then expanded for further experimentation.

### Acridine orange staining for autophagy

Formation of acidic vesicular organelles (AVOs), a morphological characteristic of autophagy [28], was quantitated by acridine orange staining. Acridine orange (1µg/ml) was added 15 min prior to collection, and cells were washed twice with phosphate buffered saline and analyzed by flow cytometry using Beckman Coulter Dako Cyan Flow Cytometer. In stained cells, acidic compartments fluoresced in bright red, quantified using FL3 (>605-635 nm), and base-line green fluorescein isothiocyanate fluorescence was measured using the FL1 channel (500-550 nm). Cells were gated on AVO high and AVO low populations. In some cases double staining with acridine orange and annexin V-APC/DAPI was performed. For double staining, after incubation with acridine orange and washing, cells were stained with annexin V-APC followed by flow cytometry. DAPI was added to the cells prior to acquisition. Apoptosis was measured on FL6-DAPI (425-475nm) and FL8-APC (655-675 nm) channels. Apoptosis was analyzed on both AVO high and AVO low population. Data were analyzed using FCS express software.

## Results

### HIF-1α is constitutively active in DLBCL cell lines under normoxia

**Figure 1:**
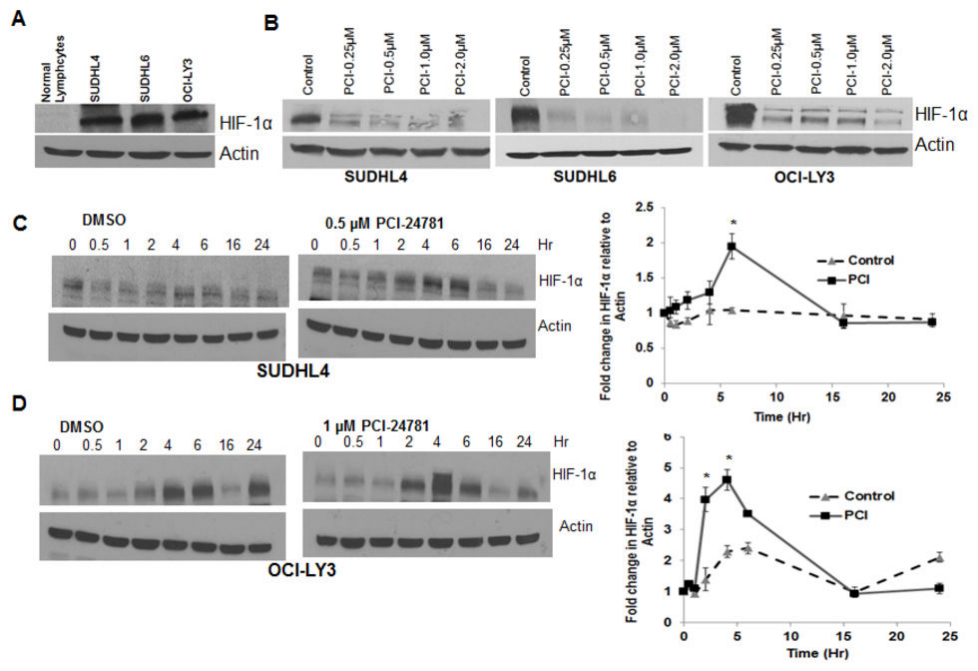
PCI-24781 modulates the expression of HIF-1α. (**A**) All cell lines as well as normal lymphocytes were subjected to lysis followed by Western blotting using HIF-1α antibody. (**B**) All cell lines were incubated with indicated concentration of PCI-24781 (PCI) under normoxia for 24 hr followed by cell lysis and western blotting using HIF-1α antibody. (**C**) SUDHL4 and (**D**) OCI-LY3 cells were incubated with or without PCI for 0-24 hr followed by western blot analysis for HIF-1α. Bands were quantified by densitometry and change in HIF1α protein over time is shown by the line graph on the right side of each blot. (* = P<0.05).

HIF-1α is stabilized by hypoxia, which is a common feature of solid tumors. However the concept of hypoxia affecting circulating cells in hematologic malignancies is not as well understood, and it is possible that there are factors other than hypoxia, are responsible for its activation in these malignancies. HIF-1α was previously found to be stabilized in non-Hodgkin’s lymphoma cells lines as well as in cells from a significant number of DLBCL patients [29]. Therefore we began by assessing the protein levels of HIF-1α in DLBCL cell lines under normoxia. Cell lysates from DLBCL cell lines SUDHL4, SUDHL6, and OCI-LY3 as well as from normal lymphocytes were subjected to Western blotting using HIF-1α antibody. As shown in Figure 1A, all the cell lines showed significant expression of HIF-1α protein under normoxia, while no HIF-1α protein was observed in normal lymphocytes, suggesting that HIF-1α is constitutively active in lymphoma cell lines under normoxia. 

### PCI-24781 modulates the expression of HIF-1α protein

Many studies have evaluated the ability of small molecules to repress HIF-1α, and several HIF inhibitors have been reported [30-34]. Although HDACI act at a wide range of targets, studies have linked their antiangiogenic and antitumor effects to HIF inhibition [35]. In our studies, treatment of cells with PCI-24781 for 24 hr. caused a significant decrease in HIF-1α protein levels (Figure 1B). The reduction in HIF-1α protein was observed after 16 hr. incubation, yet shorter incubations (0-6 hr.) resulted in a paradoxical increase in HIF-1α protein in SUDHL4 (Figure 1C) and OCI-LY3 (Figure 1D) cell lines when treated with 0.5 µM and 1.0 µM PCI-24781 respectively. A change in HIF-1α over time has been shown by the line graph next to each blot. HIF-1α protein was constitutively increased in lymphoma cells without PCI-24781 treatment, but the HDACI caused a further increase in HIF-1α protein, compared with untreated cells during the 0-6 hr period. The early increase in HIF-1α protein was sustained in control cells over 16 hr., whereas it decreased in cells treated with PCI-24781. The high expression of HIF-1α and its further increase in response to PCI-24781 at early time points to the extent that HIF-1 promotes growth and survival in DLBCL cells may promote lymphoma progression. 

### Suppression of HIF-1α enhances PCI-24781-induced apoptosis

**Figure 2:**
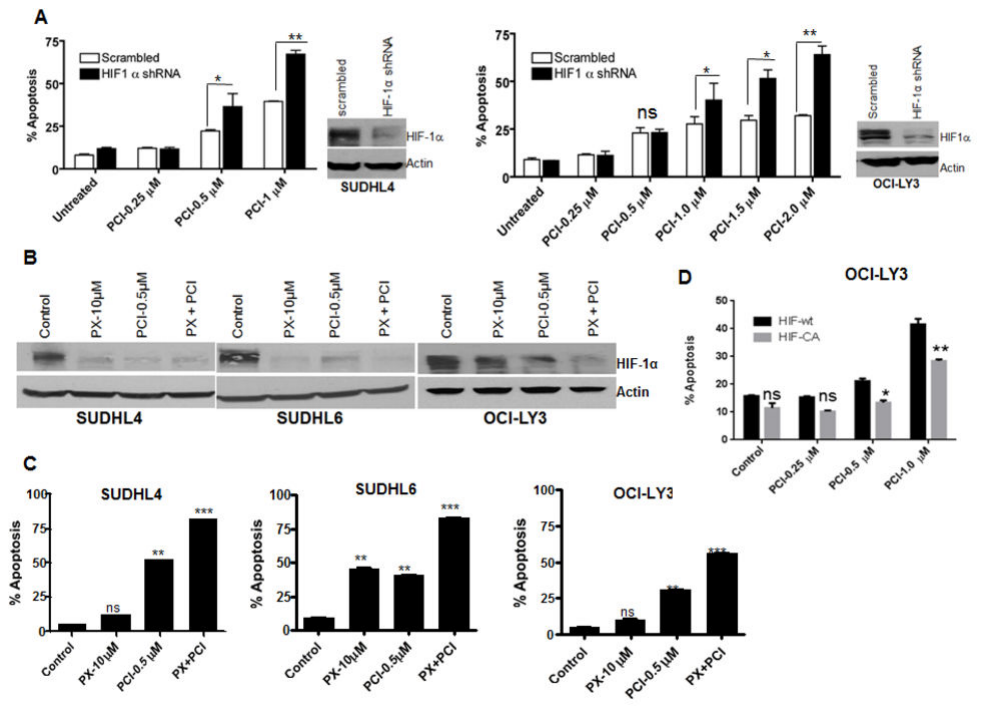
Knock down of HIF-1α enhances PCI induced apoptosis. (**A**) HIF-1α was knocked down in SUDHL4 and OCI-LY3 cell lines. Cells were transduced with scrambled or HIF-1α shRNA by spin infection using GIPZ lentivirus system.  After selection in puromycin for 2 weeks, cells were subjected to Western blotting to check the protein level and after positive selection cells were treated with indicated concentration of PCI-24781 for 48 hours, followed by annexin V/PI staining and analyzed by flow cytometry. Western blot next to each bar graph shows knock down of HIF-1α (**B**) SUDHL4, SUDHL6 and OCI-LY3 cells were incubated with indicated concentration of PCI-24781 and PX-478 either alone or in combination for 24 hr followed by cell lysis and western blotting. Actin is used as an internal control for all western blots. (**C**) SUDHL4, SUDHL6 and OCI-LY3 cells were treated with indicated concentration of PCI or PX-478 alone or in combination for 48 hr followed by annexin V/PI staining and analyzed by flow cytometry. (**D**) OCI-LY3 cells were transfected with wt-HIF-1α or mutant (P/A) HIF-1α plasmids using Amaxa Kit L and selected in neomycin for one week. After selection cells were treated with indicated concentration of PCI for 48 hr followed by AnnexinV/PI staining and analyzed by flow cytometry. (ns= not significant,. * = P<0.05; **= p<0.01; *** = P<0.001).

We have shown previously that PCI-24781 induces caspase-dependent apoptosis in lymphoma cells [36]. Since DLBCL cells have initial high expression of HIF-1α, which plays an important role in cell growth and survival, we sought to test whether suppression of HIF-1α enhances PCI-induced apoptosis. We suppressed HIF-1α in SUDHL4 and OCI-LY3 cell lines by transducing cells with lentivirus containing HIF-1α shRNA or scrambled shRNA. After selection in puromycin for 2 weeks, cells were treated with PCI-24781 for 48 hr. As shown in Figure 2A, suppression of HIF-1α enhanced PCI-24781-induced apoptosis by 2-fold in SUDHL4 and OCI-LY3 cell lines. As an alternative to shRNA suppression, we evaluated the effects of a small molecule inhibitor of HIF-1α (PX-478) on the response to PCI-24781.Treatment of cells with PX-478 in combination with PCI-24781 produced a significant decrease in HIF-1α protein (Figure 2B); which was associated with an enhanced apoptosis induced by PCI-24781(Figure 2C). By contrast, exogenous expression of constitutively active HIF-1α in cells resulted in less apoptosis in response to PCI-24781 as compared to cells expressing wild type HIF-1α (Figure 2D), confirming a role of HIF-1α in providing resistance against PCI-24781-induced apoptosis. Collectively, these data suggest that HDACI-induced apoptosis is enhanced in DLBCL cell lines by reduction of HIF-1α, either by knockdown or pharmacologic inhibition.

### PCI-24781-induced suppression in HIF-1α protein does not require proteasomal degradation

**Figure 3:**
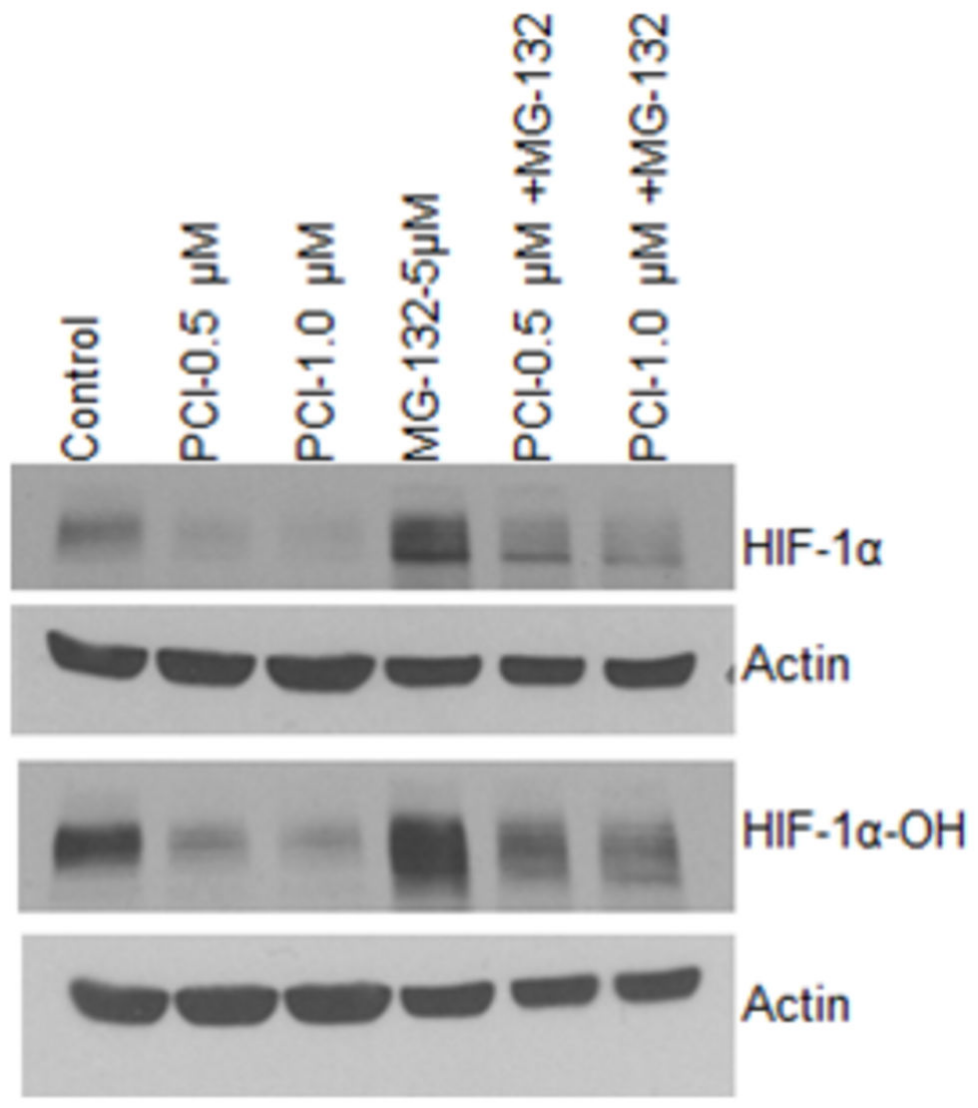
PCI-24781-induced suppression in HIF-1α protein does not require proteasomal degradation. OCI-LY3 cells were incubated with indicated concentrations of PCI-24781 or MG132 alone or in combination for 24 hr followed by western blotting using specific antibodies to HIF-1α and HIF-1α-OH.

To explore the underlying mechanisms of PCI-24781-induced suppression of HIF-1α protein, we tested whether this effect is due to increased proteasomal degradation. Under normoxic conditions HIF-1α is hydroxylated at conserved proline residues by prolyl hydroxylases allowing their recognition and ubiquitination by the VHL E3 ubiquitin ligase and rapid degradation by the proteasome [37] . To determine how PCI-24781 enhanced HIF-1α degradation, we treated OCI-LY3 cell line with PCI-24781 and a proteasome inhibitor, MG-132 and analyzed the expression of HIF-1α and hydroxylated HIF-1α protein by immunoblotting. As shown in Figure 3, co-treatment of cells with MG-132 did not inhibit the effect of PCI-24781 on HIF-1α protein or its hydroxylation (hydroxy HIF-1α), suggesting that the decrease in HIF-1α after PCI-24781 treatment is not due to increased degradation through proteasomal activity. 

### PCI-24781-induced suppression of HIF-1α is regulated by the PI3K/Akt pathway

**Figure 4:**
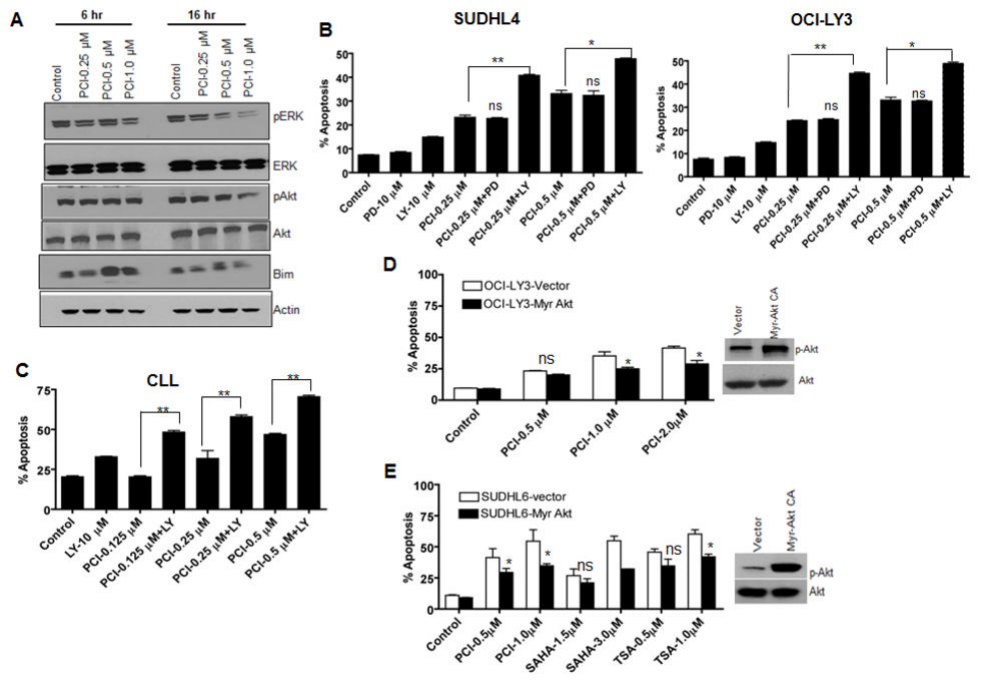
PCI-24781 induced Apoptosis is mediated by PI3K/Akt pathway. (**A**) OCI-LY3 cells were treated with indicated concentrations of PCI-24781 for 6 or 16 hr followed by cell lysis and western blotting for indicated proteins. (**B**) SUDHL4 and OCI-LY3 cells were pre-incubated with PD98059 (PD) or LY294002 (LY) for 2hr followed by 48 hr. incubation with PCI-24781. Cells were stained with AnnexinV/PI and analyzed by flow cytometry. (**C**) Malignant B- cells obtained from a CLL patient blood sample using Histopaque were incubated with PCI for 44 hr followed by Annexin V/PI staining and analyzed by flowcytometry. (**D**) SUDHL6 and OCI-LY3 cells were transfected with Myr-Akt or empty vector using Amaxa kit V and L respectively followed by selection in neomycin for one week. OCI-LY3 Cells were analyzed by western blotting for constitutive activation of AKT by western blotting and incubated with PCI-24781 for 48 hr followed by annexinV/PI staining and analyzed by flow cytometry. (**E**) SUDHL6 expressing constitutively active AKT (Myr-Akt) and wt -AKT were incubated with indicated concentrations of PCI-24781, SAHA and TSA for 48 hr followed by Annexin V/PI staining and analyzed by flow cytometry. (ns= not significant, * = P<0.05; **= p<0.01; *** = P<0.001).

We next investigated the factors that might regulate HIF-1α in lymphoma cells under normoxia. As MEK/ERK [38] and PI3K/AKT pathways [39] have been implicated as upstream activator of HIF-1α, it is conceivable that the HIF-1α suppressive effects of PCI-24781 in DLBCL cells are attributable to an interaction of the drug with an upstream kinase. Therefore we examined the effects of PCI-24781 on expression of ERK (pERK and total ERK) and AKT (pAKT and total AKT) protein by Western blotting in DLBCL cell lines. Treatment of cells with PCI-24781 resulted in a significant decrease in phosphorylation of AKT (Ser 473) and ERK proteins (Figure 4A). An increase in the proapoptotic protein Bim was also observed suggesting induction of apoptosis in association with the decrease in Akt or ERK activation. 

To further understand the effect of MEK/ERK and AKT pathways in PCI-24781-induced apoptosis, we treated cells with PCI-24781 in combination with PD98059 (MEK inhibitor) or LY294002 (PI3K inhibitor) for 48 hr. and measured apoptosis using annexinV/PI. Treatment of cells with LY294002 enhanced PCI-24781-induced apoptosis while PD98059 had no effect (Figure 4B), suggesting that the PI3K/AKT but not the MEK/ERK pathway is required for PCI-24781-induced apoptosis. Similar results were obtained when primary chronic lymphocytic leukemia (CLL) cells obtained from CLL patients were treated with PCI-24781 and LY294002 alone or in combination. Co-treatment of CLL cells with LY294002 enhances PCI-24781-induced apoptosis (Figure 4C). To confirm the role of AKT in PCI-24781 induced apoptosis we transfected cells (OCI-LY3 and SUDHL6) with vector or constitutively active AKT (Myr-Akt); after subsequent treatment with PCI-24781, apoptosis was assesed by annexin V/PI staining. As shown in Figure 4D, cells transfected with Myr-Akt showed less apoptosis as compared to cells transfected with vector alone. Similar results were obtained when SUDHL6 cells expressing Myr-Akt were treated with PCI-24781 and two other HDACI, such as SAHA (suberoylanilide hydroxamic acid) or TSA (Trichostatin A) (Figure 4E). These results suggested that the effect of AKT activation on apoptosis is not specific to PCI-24781 but rather a phenomenon common to multiple HDACI.

**Figure 5:**
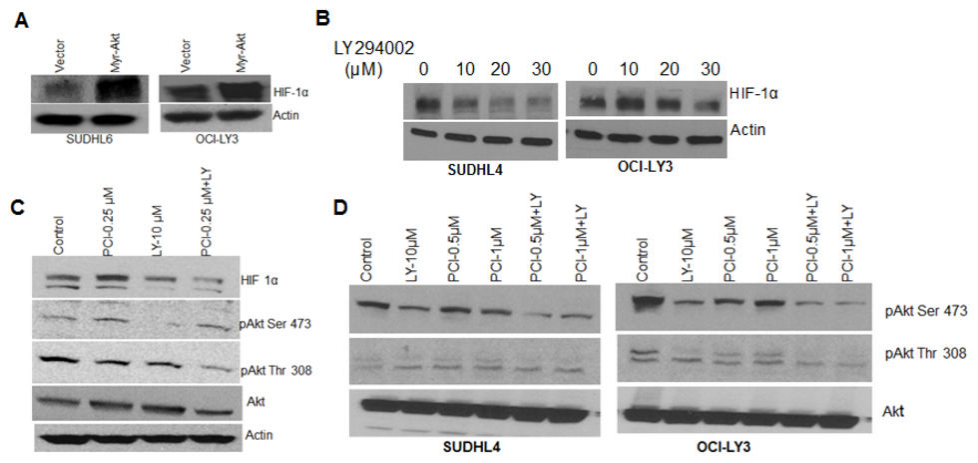
PI3K/AKT pathway in HIF-1α regulation. (**A**) To check the expression of HIF-1α in SUDHL6 and OCI-LY3 cells expressing Myr-Akt and wt-Akt, cells were subjected to lysis followed by western blotting using HIF-1α antibody. (**B**) SUDHL4-and OCI-LY3 cells were incubated with indicated concentration of LY294002 (LY) for 24 hr followed by cell lysis and Western blotting using HIF-1α antibody. (**C**) Malignant cells obtained from blood sample of CLL patient were incubated with indicated concentration of PCI or LY alone or in combination for 16 hr followed by cell lysis and western blotting using specific antibodies as indicated. (**D**) SUDHL4 and OCI-LY3 cells were treated with PCI and LY either alone or in combination for 24 hr followed by cell lysis and western blotting using specific antibodies for pAkt and Akt. Actin is used as an internal control.

PI3K/AKT has already been shown to regulate HIF-1α translation [40], so we reasoned that cells expressing constitutively active AKT should show a higher level of HIF-1α protein. Thus we measured expression of HIF-1α protein in Myr-Akt transfected lymphoma cells. OCI-LY3 and SUDHL6 cells transfected with Myr-Akt exhibited a higher level of HIF-1α protein as compared to cells transfected with vector alone (Figure 5A). On the contrary, cells treated with increasing concentrations of LY294002 (LY) for 24 hr. showed dose-dependent decrease in HIF-1α protein (Figure 5B). Primary CLL cells treated with PCI-24781 or LY294002 also resulted in decreased pAKT (Thr 308) and HIF-1α, which was further decreased with the combination of the two drugs (Figure 5C). Moreover cells treated with LY294002 alone exhibited a lower protein expression of HIF-1α, suggesting that PI3K/AKT augments its expression. SUDHL4 and OCI-LY3 cells also showed further decreases in pAKT (Ser 473) following co-treatment with LY294002 as compared to PCI-24781 alone (Figure 5D). Collectively these results suggest that AKT activates HIF-1α protein expression in DLBCL cells to the extent that HIF-1 is protective and its suppression by PCI-24781 could induce apoptosis.

### PCI-24781 induces prosurvival autophagy in DLBCL cell lines

**Figure 6:**
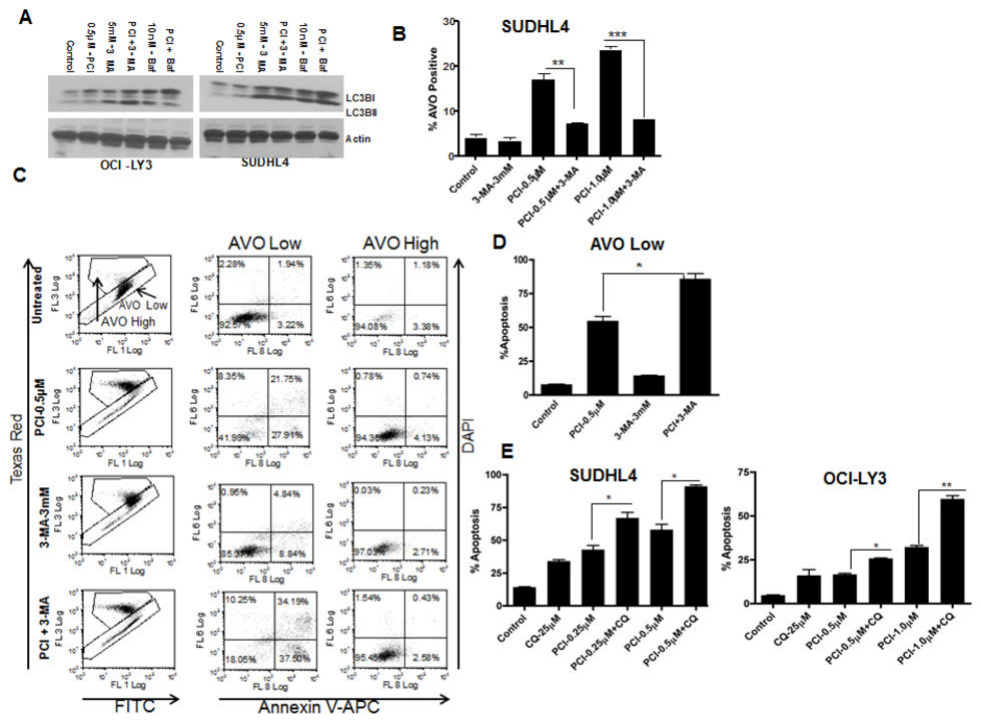
PCI-24781 induces prosurvival autophagy. (**A**) SUDHL4 and OCI-LY3 cells were treated with 0.5 µM PCI and 5 mM 3-MA or 10 nM Bafilomycin (Baf) either alone or in combination for 6 hr followed by cell lysis and western blotting for LC3B protein. Actin is used as an internal control. (**B**) SUDHL4 cells were treated with 3 mM 3-MA and indicated concentration of PCI-24781 either alone or in combination for 16 hr followed by additional incubation with 2 µg/mL acridine orange for 15 minutes. Percentage of cells representing formation of Acidic vesicular organelles was quantified by flow cytometry. (**C**) SUDHL4 cells were treated with PCI or 3 –MA either alone or in combination for 48 hr followed by further incubation with 2 µg/mL acridine orange and analyzed by flow cytometry. DAPI was added to cells prior to acquisition. Cells were gated on AVO low and AVO high population and apoptosis was measured on each population by gating on Annexin V–APC (FL8) and DAPI (FL6) positive cells. (**D**) Bar graph representation of % of apoptosis on low AVO population obtained from Figure 5 C. (**E**) SUDHL4 and OCI-LY3 cells were incubated with PCI-24781 and Chloroquine (CQ) either alone or in combination for 72 hr followed by annexin V/ PI staining and flow cytometry. (* = P<0.05; **= p<0.01).

As HDAC inhibition has been shown to induce autophagy [26], we investigated the mechanism by which PCI-24781 may induce autophagy and its role in apoptosis. DLBCL cells were treated with PCI-24781 and assayed for expression of the autophagy marker LC3B by Western blotting. PCI-24781 caused an accumulation of LC3-II. Additional treatment with the lysosomal inhibitor Bafilomycin A1 (Baf A1) or the autophagy inhibitor 3-methyl adenine (3-MA) caused a further increase in LC3-II level (Figure 6A). In Baf A1/3-MA treated cells, autophagy is inhibited before the fusion of autophagosomes and lysosomes. As a result, LC3-II aggregates on the autophagosomes, and could not be degraded through the fusion of autophagosomes and lysosomes. Therefore, in these cells, the increase of LC3-II expression indicates autophagy is suppressed.

To detect cells undergoing autophagy or formation of acidic vesicular organelles (AVO) we stained the cells with acridine orange (a lysosomal marker), which fluoresces bright red under the acidic conditions in autophagolysosomes. Treatment of cells with PCI-24781 for 16 hr caused the appearance of a population with a high AVO content indicating the activation of autophagy by PCI-24781, which was inhibited by 3-MA, (Figure 6B). To determine whether autophagy influenced the apoptosis in PCI-24781 treated cells, we examined the effect of autophagy inhibitors, 3-MA and chloroquine (CQ) on PCI-24781-induced cell death. As shown in Figure 6C (SUDHL4 cell), an enhancement of apoptosis was observed when autophagy was blocked in PCI-24781-treated cells. Cells with high AVO staining showed less apoptosis, suggesting that autophagy protects against apoptosis. Moreover, co-treatment of cells with the autophagy inhibitor 3-MA, resulted in a decrease in high AVO population and increase in low AVO cells and apoptosis as compared to PCI-24781 alone (Figure 6D). While 3-MA is primarily a research agent, another autophagy inhibitor, CQ has been clinically available as an antimalaria agent. Therefore we tested the effect of CQ on PCI-24781-induced apoptosis. Co-treatment of SUDHL4 and OCIL Y3 cells (Figure 6E) with CQ decreased the high AVO content and enhanced apoptosis. Collectively these results suggest that PCI-24781-induces autophagy in DLBCL cells, which may undermine the pro-apoptotic effects of the drug. 

### PCI-24781-induced autophagy is mediated by HIF-1α

**Figure 7:**
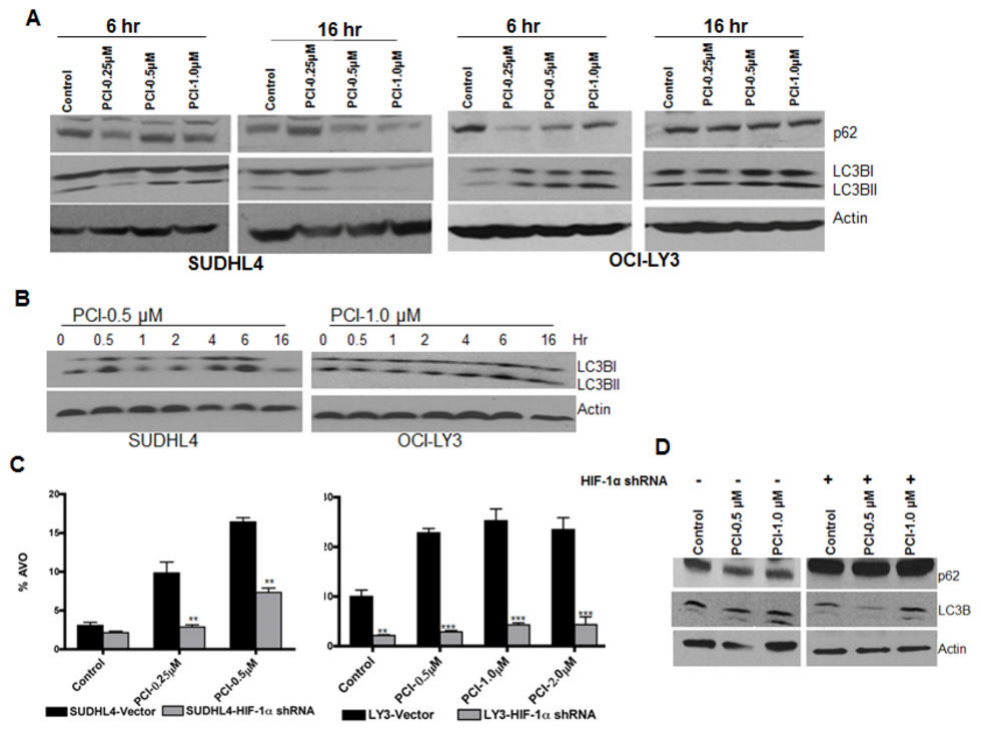
PCI-24781- Induced autophagy is mediated by HIF-1α. (**A**) SUDH4, SUDHL6 and OCI-LY3 cells were incubated with increasing concentration of PCI-24781 for 6 or 16 hr followed by cell lysis and western blotting for LC3B and p62 protein using specific antibodies. (**B**) SUDHL4 and OCI-LY3 cells were incubated with 0.5 or 1.0 µM of PCI respectively for 0-16 hr followed by cell lysis and western blotting for LC3B protein using specific antibody. (**C**) SUDHL4 and OCI-LY3 cells expressing scrambled or HIF-1α shRNA were treated with PCI for 24 hr followed by further incubation with acridine orange for 15 minute. Cells were washed twice with PBS and analyzed by flow cytomerty for the quantification of cells with high AVO population. (**D**) OCI-LY3 cells with or without HIF-1α shRNA were treated with PCI-24781 for 6 hr followed by cell lysis and western blotting for autophagy markers p62 and LC3B proteins. Actin is used as an internal control for western blotting. (* = P<0.05; **= p<0.01; *** = P<0.001).

Finally, we examined whether HIF-1α, an upstream activator of autophagy, was involved in PCI-24781-induced autophagy [41]. We initially demonstrated that PCI-24781 caused an upregulation of HIF-1α expression within 6 hr. while extended incubation for 16 hr. with the drug resulted in HIF-1α downregulation. As shown in Figure 7A, a downregulation of the pro-autophagy protein p62 and an increase in LC3B cleavage was also observed at 6hr suggesting that induction of autophagy coincides with increases in HIF-1α expression. Figure 7B shows an increase in LC3BII expression from 0-6hr after treatment with PCI-24781 in both SUDH4 and OCI-LY3 cells. 

To better understand the functional role of HIF-1α in PCI-24781-induced autophagy, we suppressed HIF-1α in SUDHL4 and OCI-LY3 cells using HIF-1α shRNA and treated them with PCI-24781 for 6hr and then assessed the autophagic response. Cells with HIF-1α suppression showed no evidence of autophagy in both cell lines as compared to control cells treated with PCI-24781 (Figure 7C). To determine the effect of PCI-24781 on autophagy in cells with HIF-suppression, immunoblots were performed to assess the expression of autophagy proteins p62 and LC3B. As shown in Figure 7D, HIF-1α knockdown cells showed minimal changes in the expression of p62 and LC3B cleavage following PCI-24781 treatment while control cells showed a decrease in p62 expression and increase in cleavage of LC3B protein. Together these studies support the notion that PCI-24781-induced autophagy is at least partially regulated by HIF-1α in DLBCL cells.

### PCI-24781 induces AMPK activation

**Figure 8:**
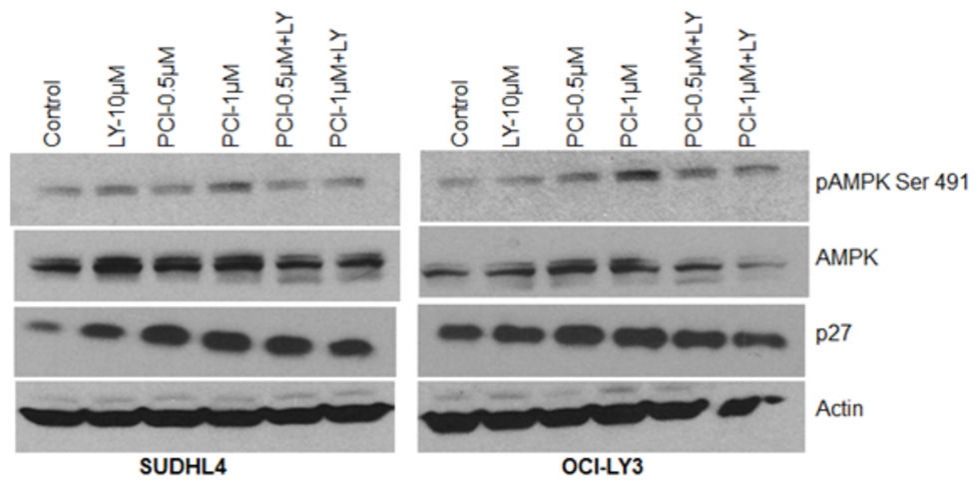
PCI-24781 induces AMPK activation. SUDHL4 and OCI-LY3 cells were treated with indicated concentration of PCI or LY alone and in combination for 24hr followed by western blotting using specific antibodies. Actin is used as an internal control.

In addition to HIF-1α, another important player in autophagy is 5’-AMP activated protein kinase (AMPK) [42]. AMPK, which is activated by AMP and ADP, functions as an intracellular energy sensor and regulates metabolism and cell proliferation. AMPK activates p27 phosphorylation at Thr198 resulting in the stabilization of p27, which permits cells to survive metabolic stresses through autophagy [43]. In our studies, AMPK activation, mediated by a decrease in pAkt, occurs in DLBCL cells treated with PCI-24781. As shown in Figure 8, both LY294002 and PCI-24781 induced AMPK and p27 activation in SUDHL4 and OCI-LY3 cell lines. However the combination of PCI-24781 and LY294002 did not show any further change in pAMPK and p27 protein as compared to either agent alone. These studies suggest that PCI-24781 may induce AMPK mediated autophagy which is independent of HIF-1α.

## Discussion

The cellular response to hypoxia is mediated in part by HIF-1α, which is key regulator of cellular adaptation to hypoxic stress. In addition to hypoxia, other physiological and pathological factors, such as cytokines, growth factors, activated oncogenes and inactivated tumor suppressors can activate HIF-1α primarily by stimulating its mRNA expression or translation [44-46]. HIFs constitute a common link among hypoxia, chronic inflammation [47,48] by induction of Toll like receptors [49], metabolic adaptation [50], and tumor progression during cancer development. A role of HIF1 has also been reported in mediating upregulation of histone demethylases [51]. Clinical studies have shown that HIF-1α overexpression correlates with advanced disease stages and poor prognosis among cancer patients [7,52]. Preclinical studies have demonstrated that HIF-1α inhibition suppresses angiogenesis and tumor growth and its suppression enhances treatment outcomes when used in combination with chemotherapeutic agents and radiation [53,54]. A growing number of HIF-1α inhibitors, including HDAC inhibitors have been identified [35]. HDAC inhibitors have been shown to decrease HIF-1α in VHL )von Hippel-Lindeau (VHL) protein) dependent [55], or VHL-independent manner [17].

We evaluated the complex biologic and molecular activity of PCI-24781, a pan HDAC inhibitor in DLBCL cell lines and primary malignant cells. We demonstrate that HIF-1α is constitutively expressed under normoxia in various DLBCL cell lines and that HDACI inhibits HIF-1α protein accumulation in these cells. However, the inhibition of HIF-1α occurs only after 16hr treatment, whereas at earlier times HDACI induces accumulation of HIF-1α (an early response). This initial increase in HIF-1α protein accumulation could be due to reactive oxygen species (ROS) generation induced by PCI-24781, as studies by our group and others have shown that HDACI induces ROS [36,56] which may play an important role in promoting HIF-1α stabilization [57,58].

We first investigated the underlying mechanism by which, PCI-24781, downregulates HIF-1α protein expression. Our data show that suppression of HIF-1α protein by HDACI was not due to proteasomal degradation as co-treatment of cells with the proteasome inhibitor, MG-132 did not prevent the decrease in protein levels after treatment with PCI-24781. In the DLBCL cells hypoxia was not a factor promoting HIF-1α stabilization. However, other intracellular signaling pathways can promote the accumulation of HIF-1α by enhancing its translation. Phosphatidylinositol 3-kinase (PI3K) /AKT is one mechanism by which HIF-1α translation and thus protein expression could be increased in DLBCL. Inhibition of PI3K/AKT pathway, is sufficient to decrease normoxic HIF-1α protein levels [59-61]. In our study, HDACI suppressed constitutive activation of AKT and decreased HIF-1α protein abundance. Moreover, constitutively active AKT could increase basal expression levels of HIF-1α protein in DLBCL cells. Therefore suppression of HIF-1α by HDACI appears to be mediated by suppression of the PI3K/AKT pathway. 

Cells exposed to hypoxia or metabolic stress, undergo autophagy during which macromolecules and cytoplasm are sequestered in autophagosomes for lysosomal degradation. Autophagy initially prevents cancer cell survival but once a tumor develops, autophagic catabolism promotes cell survival by providing nutrients under hypoxic and metabolic stresses [62]. Previous studies have shown that HIF-1 regulates autophagy-associated factors, like BNIP3 [41] but, whether the response is cytoprotective remains undetermined. We hypothesized that early accumulation of HIF-1α following HDACI treatment would promote autophagy as a cytoprotective adaptive mechanism. 

Several cancer therapies including HDACI have been shown to induce autophagy in cancer cells [26,42,55]. As with other anticancer therapies, HDACI-induced autophagy appears to act as a pro-survival mechanism to counteract cytotoxic activity. Autophagy may delay the onset of apoptosis during HDACI treatment, which may compromise its therapeutic efficacy. We observed that PCI-24781 induces autophagy in DLBCL cells and its inhibition by 3-MA or CQ enhances PCI-24781-induced apoptosis. Our data also indicate that HDACI induces HIF-1α-mediated autophagy and that the autophagy response is inhibited by suppressing HIF-1α protein expression. Tumor cells might depend on HIF-1α to sustain viability by promoting autophagy during tumorigenesis as well as during therapy. Importantly, our findings also show that inhibition of autophagy by CQ or 3-MA, sensitizes DLBCL cells to PCI-24781-induced apoptosis which may be important in overcoming resistance *in vitro* and possibly *in vivo*. While HDACI are being combined with multiple anticancer agents [21], only a few promising strategies targeting HIF-1α in lymphoma [63] are currently under development. Based on our data, HIF-1α suppression should be considered as a potential mechanism contributing to the ability of HDACI to inhibit cancer cell growth.

Besides HIF-1α, other factors such as AMPK and p27 might play a role in PCI-24781 induced autophagy resulting in reduced efficacy of the drug. In DLBCL cells treated with PCI-24781, we observed an elevated AMP-activated protein kinase (AMPK) signaling and an increase in p27 protein. Activation of AMPK might occur due to suppression of AKT activity, as AMPK activity and AKT signaling are mutually antagonistic [64,65]. Whether HDACI induced activation of AMPK and p27 plays a role in autophagy remains unclear. Further studies are needed to understand the role of AMPK and p27 in PCI-24781 induced autophagy in DLBCL cells.

**Figure 9:**
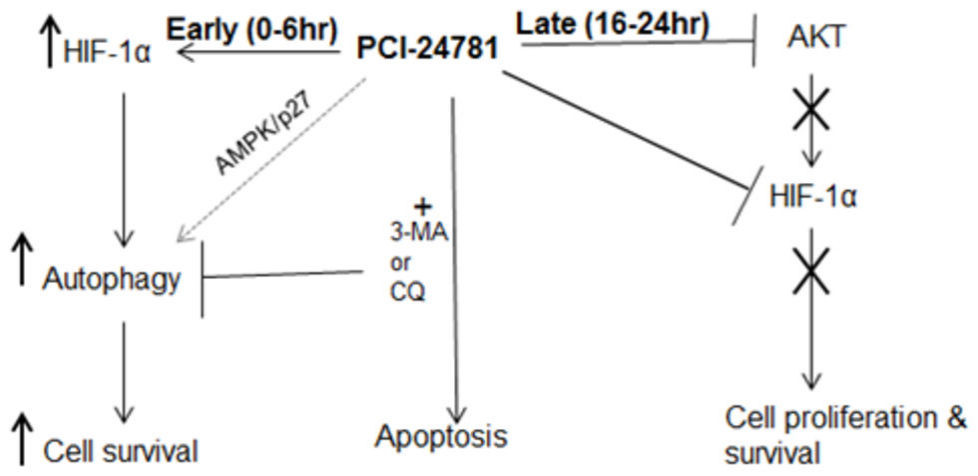
Schematic representation of the role of PCI-24781 in regulation of HIF-1α and autophagy. PCI24781 enhances accumulation of HIF-1α in first 6hr of incubation resulting in induction of autophagy and cell survival (**early**). Longer incubation with PCI-24781 caused suppression of AKT activity and HIF-1α protein expression (**Late**) resulting in apoptosis. Autophagy inhibitors enhance PCI-24781 induced apoptosis. PCI-24781 might also activate AMPK and p27 mediated autophagy.

Taken together, our studies clearly uncover a novel function of HIF-1α in HDACI-induced autophagy and show its impact on cell survival (Figure 9). Our rationale to target HIF-1α and autophagy via a combination of HDACI and autophagy inhibitors represents a novel therapeutic option for the treatment of lymphoma.
